# Clinical Evaluation of Root Canal Shaping Ability of a Stainless-steel File System Compared to Two Nickel-titanium Single-file Systems

**DOI:** 10.4317/jced.62012

**Published:** 2024-11-01

**Authors:** Alaa O. Mais, Amr M. Abdallah, Essam Osman, Hatem A. Alhadainy

**Affiliations:** 1 Division of Endodontics, Department of Restorative Sciences, Faculty of Dentistry, Beirut Arab University, Lebanon; 2 Division of Endodontics, Department of Restorative Dentistry, Faculty of Dentistry, Alexandria University; 3 Dental Biomaterials, Faculty of Dentistry, Alexandria University; 4 Division of Endodontics, Department of Dentistry, University of North Carolina, Chapel Hill, USA. On leave from Tanta University, Egypt

## Abstract

**Background:**

Root canal shaping is an important phase in endodontic treatment to preserve the integrity of root structures. This clinical study used cone-beam computed tomography (CBCT) to assess the shaping ability of Tornado compared to WaveOne (WO), and OneShape (OS) rotary systems.

**Material and Methods:**

MesioBuccal canals of lower molars with curving angles ranging from 15° to 45° were used in 30 patients to evaluate the apical transportation and centering ability of Tornado, WO, and OS. The canals were divided into 3 groups according to the instrumentation system (n=10), which corresponded to the systems that were employed. Centering ratio and canal transportation were assessed at 2, 5, and 8 mm from the apical foramen using CBCT. Statistical analysis was conducted using the Kruskal-Wallis test and Mann-Whitney test at p-value = 0.05.

**Results:**

All tested levels showed no statistically significant difference in transportation and centering ability (*P*>0.05). The Tornado file preserved the original canal curvature with no statistical significance compared to WO files and OS.

**Conclusions:**

The tornado file system was proven to provide an accepted quality of root canal shaping compared to WO and OS rotary systems. All systems maintained the original canal anatomy with appropriate centralization and no canal transportation.

** Key words:**Canal Centering Ability, Canal Transportation, Cone-beam Computed Tomography, Nickel-Titanium Files, Tornado Rotary System.

## Introduction

The objectives of root canal preparation are to remove any pulp tissues, eradicate microorganisms and their byproducts, and preserve the integrity of root structures using a chemo-mechanical technique that combines mechanical instrumentation with canal irrigation (1). Continuous tapering preparation with uniform dentin removal and minimal wear on the root structures is essential for preserving the original canal anatomy through consistent instrumentation of all canal surfaces (2). Procedural mishaps such as zipping, ledging, perforations, and apical transportation should be avoided to obtain successful endodontic treatment (3). However, preventing these unfavorable mishaps is challenging, particularly in severely curved canals (2). Therefore, a high centering ratio and less canal transportation are essential to provide proper canal shaping without unduly weakening the root structures (4). Apical transportation causes loss of the working length, and transportation results in excessive or eccentric shaping and improper canal straightening with possible excessive dentin removal, elbow, biomechanical defects, and root fracture (5). The quality of root canal shaping can be assessed with Cone beam computed tomography (CBCT) imaging that provides quantitative and qualitative values for root canal geometry in three dimensions. CBCT has been recommended as a non-destructive method for the assessment of changes in root canals before and after instrumentation (6). Nickel-titanium (NiTi) instruments provide excellent flexibility, super-elasticity, and shape memory (7). However, canal preparation with NiTi instruments may result in microcracks in dentin (8), and torsion and cyclic fatigue may inevitably cause instrument fracture. Therefore, a new stainless-steel (SS) system (Tornado file system, MBI Tornado, France) was introduced and the manufacturer claimed it to be more flexible than NiTi instruments with superior qualities. The Tornado system consists of six files which are one file for canal orifice opening (18 mm) and five files for canal preparation (25 mm). Every file is made up of SS wires wrapped on a cable core. It splits into an upper section with three layers and an apical section with a bi-layer structure. The rough surface is achieved by metal particle injection technology. The file consistently tapers down to 4% with its apical 0.5 mm edge sharpened at a 45° angle, producing a passive tip that is non-cutting. A specialized motor that automatically adapts to load and runs at a maximum speed of 6500 rpm is connected to these instruments through friction (9). High-speed rotation influences the fluid dynamics within the canal and improves irrigation efficiency (10).

Single-file rotary systems are frequently used and proven to have good shaping ability; the issue that made these systems a gold standard for the ability to shape the root canal.

WaveOne (WO) file (Dentsply Maillefer, Ballaigues, Switzerland) is a single reciprocating file system with a heat-treated NiTi Memory Wire (M Wire). It utilizes the balanced force technique developed by Roane which proved to have more flexibility and fatigue resistance (11,12). Another single-file system is the OneShape (OS) file (Micro-Mega SA, Besançon, France) that utilizes a conventional, continuous rotational motion. It has asymmetric cross-sectional geometry, and traveling waves of motion that are produced along the file’s active region (13).

Limited studies have evaluated the shaping ability of the Tornado system when compared to NiTi Single-file rotary systems. This study used CBCT to compare the apical transportation and centering capabilities in curved root canals instrumented with a single Tornado rotating stainless-steel system, a single WO reciprocating file, and a single OS rotary file.

## Material and Methods

-Selection of Patients and Ethical Approval

The protocol of this randomized clinical study received ethical clearance from the Institutional Review Board. The study population was patients from the Endodontic Clinic at Beirut Arab University who sought nonsurgical root canal therapy. The study sample of 30 patients was randomly selected with two separate mesial canals in their lower molars. The inclusion criteria were based on the work of Deepak *et al*. (14). The canals should have distinct apical foramen, mature apices, and no resorption or fractures. Canal curvature was measured using the Schneider method, mesial canals should have a curvature within the range of 15° to 45° (15). The study excluded patients who were younger than 16 or older than 65, had diabetes, immune-compromising conditions, or previously had dental work done on the investigated tooth (16). After being informed of the potential dangers and discomfort as well as the potential advantages, patients who met the inclusion criteria signed informed consent for the treatment protocol which was authorized by Beirut Arab University’s Institutional Review Board (IRB). All patients acknowledged that they were involved in dental research and agreed and permitted the publication of the results. Every patient was reviewed and assessed through appropriate history-taking, clinical examinations, and pre-operative digital radiography. The patient’s medical history, including past dental and medical records, major complaints, and demographic information, was acquired, and thoroughly documented. A CBCT (Kodak 9000C) with an 80 kV, 4 mA, 51 × 51 mm field of view, and 0.1/voxel (mm) size was used to scan the teeth under investigation with an axial slice thickness of 0.1 mm at 2, 5, and 8 mm from the canal’s apex.

-Study groups:

Following the computer’s recording, the patients were randomly divided into three equal groups according to the file system used for root canal instrumentation with 10 patients in each group.

Group 1 (G1): Rotary SS Tornado system (MBI Tornado, France).

Group 2 (G2): Reciprocating NiTi WO system (Dentsply Maillefer, Ballaigues, Switzerland).

Group 3 (G3): Rotary NiTi OS system (Micro-Mega SA, Besançon, France).

-Canal preparation

**Figure 1:**
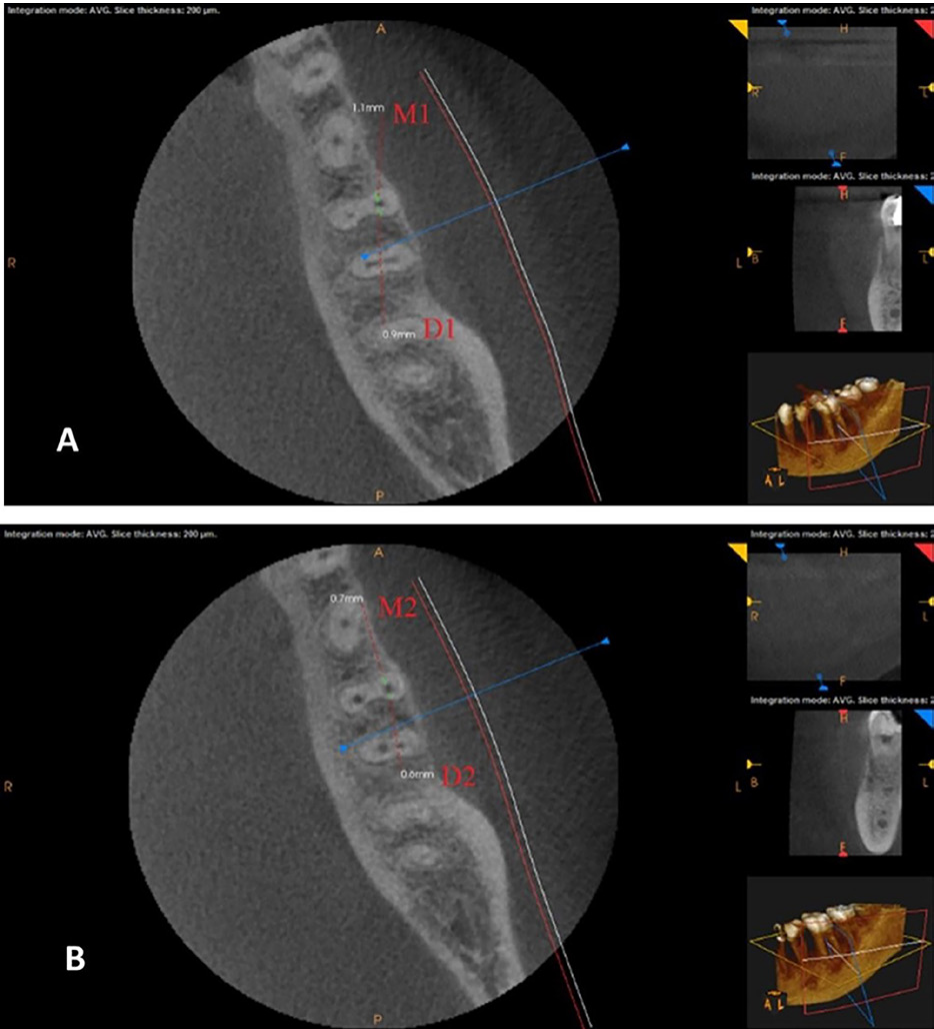
Points of measurements used for determining canal shaping in MB canal for pre-instrumentation (A) and post-instrumentation (B) CBCT images.

**Figure 2:**
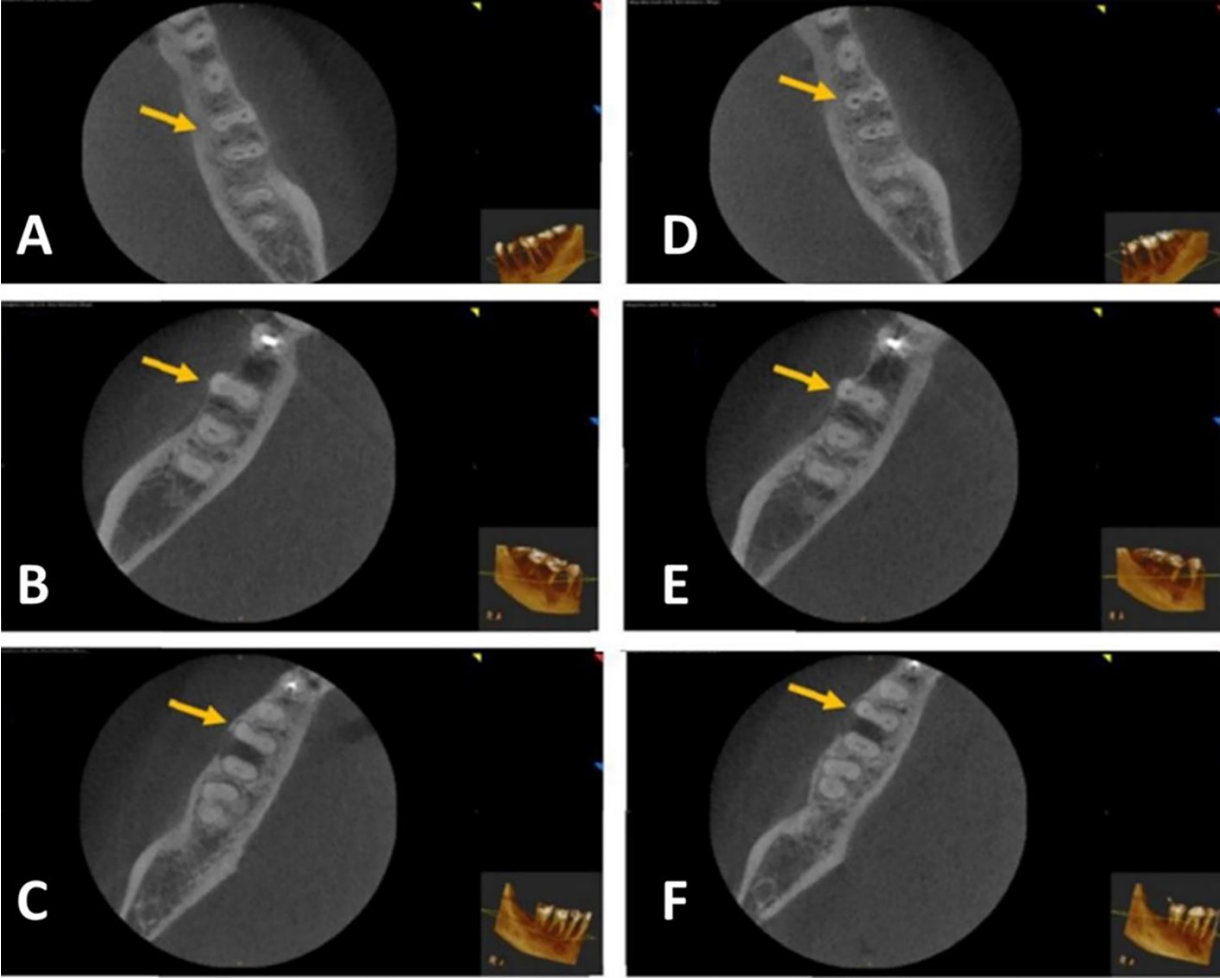
CBCT images for pre-instrumentation (A, B, C) and post-instrumentation (D, E, F) with Tornado (A, D), WaveOne (B, E), and OneShape systems (C, F) at 8 mm from the apex.

Following appropriate anesthesia and isolation, access cavities were completed using a round bur and Endo Z bur. A SS K-file #10 (MANI, Vietnam) was used for apical patency, an apex locator (DENTA PORT ZX, Japan) was used to identify the working length (WL), and a periapical X-ray was used to confirm it. A glide path (RC prep, Premier Dental, USA) was used on a size 15 K-type file for canal patency. Then, 2 ml of a 2.5% sodium hypochlorite (NaOCl) solution was used to irrigate the root canals followed by 3.0 mL of 17% ethylene-diamine-tetraacetic acid (EDTA) (NEXABIO, Korea) for one minute, and then 1.3% NaOCl was used (14). The Tornado file system (G1) was used in a special handpiece at 6500 rpm (MIB handpiece). Root canals in eight teeth were completed with a 4% taper and an apical diameter of 0.25 mm. The canals in G2 (WO system) were instrumented using a file system taper of 0.08 in apical 3 mm, with a tip diameter of 0.25 mm. in a torque control endodontic handpiece (X smart plus with reciprocation mode). The OS file system (G3) was used in a torque control endodontic handpiece (X smart plus rotational speed 250 rpm.). All instrumentation was completed following the manufacturer’s instructions and every tooth was imaged using the same methodology as the pre-instrumentation images. Cross-sectional planes of images were taken at 2, 5, and 8 mm from the apex of the mesiobuccal (MB) root and were analyzed for transportation and centering ability (17). Figure 1 shows pre- and post-instrumentation CBCT images with markings of measurement points for determining canal transportation and centering ratio in the MB canal. All images were saved on the computer for shaping analysis. Figure 2 shows CBCT scan images before and after instrumentation of the tested systems at 8 mm from the apex.

-Assessment of canal transportation

Canal transportation was evaluated by measuring the shortest path between the mesial and distal borders of the root and the periphery of pre-instrumentation images, the outcome was compared with the post-instrumented image measurements, and the amount of canal transportation in the mesial-distal direction was assessed. Every value was measured by two assessors, and a mean value was determined (18). Canal transportation was assessed using the following formula:

Canal transportation = (M1–M2)–(D1–D2).

where “M1” denotes the shortest distances from the mesial edge of the root to the mesial edge of the pre-instrumented canal, and “M2” refers to the shortest distances from the mesial edge of the root to the mesial edge of the post-instrumented canal. “D1” and “D2” refer to the shortest distances from the distal edge of the root to the distal edge of the pre-instrumented canal and post-instrumented canal respectively. A result of zero indicated no transportation, a result of a negative value indicated transportation in the distal direction, and a result of a positive value indicated transportation in the mesial direction.

-Assessment of centering ability

The instrument’s ability to remain centered in the canal is shown by the mean centering ratio. It was computed using the following ratio for every section:

Centralization ability ratio = (M1- M2) / (D1- D2) or (D1-D2) / (M1-M2).

The formula was chosen so that the numerator would be the lowest of the results obtained by the difference. A score of 1.0 indicated complete centralization. The instrument’s ability to sustain itself in the canal’s central axis was indicated by a value that was closer to zero. Finally, all of the teeth in the three groups were obturated with gutta-percha (DiaDent, Korea) sealer (Resin sealer) (META BIOMED, Korea) using the same obturation technique (Lateral condensation technique).

-Analytical Statistics 

The values of the pre- and post-instrumentation were recorded in an Excel spreadsheet. The Statistical Package for the Social Sciences (SPSS) software (SPSS 22, SPSS Inc, Chicago, IL) was used to perform the statistical analysis. Means and standard deviations were computed for all groups. A normality test for the obtained data was performed to determine the distribution of data. Since the data did not follow a normal distribution, the significant difference between groups was assessed using the Kruskal-Wallis test. Mann-Whitney test was performed to test the difference between the two groups. All statistical tests were performed at a *p-value* of 0.05.

## Results

The age of 30 patients ranged from 16 to 65 with a mean age of 35.7 ± 10.1 years. The MB root canal was used to assess the canal transportation and central ability. The data obtained from the CBCT images showed the least cumulative canal transportation in millimeters for the WO file system (0.406 ± 0.157) followed by the OS file system (0.063 ± 0.307) and the Tornado file system (-0.087 ± 0.201). The negative value of the Tornado file system indicated that canal transportation was in the distal direction, and the positive values for WO and OS systems indicated mesial canal transportation. Statistical analysis revealed no significant difference in canal transportation between the tested systems (*p*≥ 0.05). Canal transportation at 2,5 and 8 mm from the apex in the MB canal for the tested file system and the *p-value* of the Kruskal-Wallis are presented in  Table 1.

The data obtained from the CBCT images showed a total centering ratio of more than 1,0 for all groups. The Tornado file system showed the highest ratio of 2.466 ± 0.669 mm/mm followed by the OS file system (2.080 ± 0.783 mm/mm) where the WO showed the lowest ratio of 1.912 ± 0.806 mm/mm. Statistical analysis revealed no appreciable variations in centering ability between the tested systems (*p*≥ 0.05). The mean and standard deviation (SD) of centering ability values for all groups and the *p-value* of the Kruskal-Wallis test are presented in  Table 2. At every tested root level, there was no statistically significant difference observed in the canal transportation and centering ability of the three systems (*p* > 0.05).

## Discussion

The primary goals of endodontic therapy are to completely eradicate all microorganisms, tissue fragments, and their byproducts while creating a sufficiently shaped canal to allow proper obturation. (19). The current study assessed the centering ability and transportation for Tornado systems compared to WO and OS in the MB canal of mandibular molars. Because of their slender and curved nature in two planes, mandibular molars with acutely curved mesial roots were selected because they present a high level of instrumentation difficulty (20). It has also been confirmed that all preparation methods and instruments tend to alter the path of the curved root canal (21). CBCT imaging of the root canals was used to assess canal transportation and centering ability before and after instrumentation.

CBCT analytical method was found to be highly accurate in determining the centering ability of a variety of rotary systems (3). Furthermore, its nondestructive nature has been advocated. The degree of canal transportation and centering ability at various distances (2mm, 5 mm, and 8 mm) from the apex was assessed using the formula developed by Gambill *et al*. in 1996 (18). Considering that this study prepared highly curved canals, its clinical relevance is still debaTable because the maximum variance in canal straightening was only 0.4 mm. It follows that the original canal curvature was effectively preserved by all three of the instruments under study.

Numerous NiTi rotary systems have been added to endodontic instruments since the early 1990s. Single file (NiTi) rotary systems were introduced to save time for biomechanical preparation and decrease associated instrumentation failures, therefore, they are now widely accepted in the clinical setting (22). There are two applications for these systems:

Reciprocal motion and continuous rotation. OS instruments’ working parts come in a variety of cross-sectional designs and pitch lengths. When rotating continuously, this design helps to eliminate the binding and threading of this file (23). The reciprocating motion helps to reduce the taper lock issue by frequently changing the direction of rotation and reducing the flexural and torsional stresses on the instrument (22). Apical canal transportation can happen during the canal preparation process because of the instrument’s propensity to remove the outside curve of the canal’s wall structure to return to its original linear shape (24). Wu *et al*. (25) noted that when the apical transportation is more than 300 μm, the sealing of the obscuration is adversely affected. Consistent with earlier research, no statistically significant variations were found for the root canal parameters assessed in this investigation (22,26-27). Reciproc, WO, and OS were found to maintain the original curvature of highly curved canals in a previous study involving extracted teeth (28). Using CBCT imaging to assess root canal transportation, Capar *et al*. (27) found that Reciproc, WO, and OS all maintained the root canal’s curvature and produced comparable canal transportation during the mesial canal preparation of lower molars. However, despite manufacturing differences, no statistically significant difference was found in terms of centering ability and transportation with the Tornado file system in our study. This unique rotary SS file has a special manufacturing design that is distinguished by an apical bi-layer structure with good flexibility for extremely curved canals. The upper section is composed of a three-layer structure that generates the vertical force necessary to drive the file into the canal (9). The manufacturer claimed that this SS file has a lower breakage rate and a lower risk of dentin cracking compared to NiTi files. The Tornado file adapts itself to any canal shape and maintains the canal anatomy even with irregular cross-sections. The smooth, polished surface of the file edge guides it into the canal with symmetric removal of the canal dentin. It showed greater cleanliness than Pro-Taper Universal system (29).

In addition to differences in demographic variables of the included subjects, the small sample size was one of the primary limitations of the current study. Consequently, it is advised that larger sample sizes be used in subsequent research projects. Ultimately, the null hypothesis is accepted because there were no discernible differences between the three rotary systems in our study in terms of transportation or centering ability.

## Conclusions

Tornado files, WO, and OS are equally appropriate for root canal instrumentation because they maintained the original anatomy of the canal and showed no significant variations in canal transportation or centralization. The Tornado system is among the safest tools for instrumenting curved root canals.

## Figures and Tables

**Table 1:** Mean and standard deviation (SD) of canal transportation values (mm) for the tested three systems and p-value of Kruskal-Wallis test.

	Tornado		WaveOne		OneShape		P-value
	Mean	SD	Mean	SD	Mean	SD	
2 mm	-0.025	0.103	0. 406	0.069	0.012	0.112	0.662
5 mm	-0.037	0.052	0	0.031	0.020	0.103	0.474
8 mm	-0.025	0.046	0	0.057	0.031	0.092	0.299
Total	-0.087	0.201	0.406	0.157	0.063	0.307	0.123

**Table 2:** Mean and standard deviation (SD) of centering ability values (mm) for the tested three systems and p-value of Kruskal-Wallis test.

	Tornado		WaveOne		OneShape		p-value
	Mean	SD	Mean	SD	Mean	SD	
2 mm	0.738	0.233	0.582	0.177	0.645	0.225	0.298
5 mm	0.832	0.236	0.622	0.264	0.686	0.274	0.272
8 mm	0.895	0.199	0.707	0.364	0.749	0.282	0.425
Total	2.466	0.669	1.912	0.806	2.080	0.783	0.342

## Data Availability

The datasets used and/or analyzed during the current study are available from the corresponding author.
